# The genetic predisposition increases the chances of schoolchildren maintaining higher adiposity levels after three years

**DOI:** 10.1186/s12887-023-03846-0

**Published:** 2023-02-03

**Authors:** Éboni Marília Reuter, Cézane Priscila Reuter, João Francisco de Castro Silveira, Ana Paula Sehn, Pâmela Ferreira Todendi, Andréia Rosane de Moura Valim, Javier Brazo-Sayavera, Elza Daniel de Mello

**Affiliations:** 1 Department of Health Sciences, University of Santa Cruz do Sul (UNISC), Av. Independência, 2293; Bairro Universitário, Santa Cruz do Sul, Rio Grande do Sul 96816-501 Brazil; 2 Graduate Program in Health Promotion, University of Santa Cruz do Sul, Santa Cruz do Sul, Brazil; 3 Graduate Program in Human Movement Sciences, Federal University of Rio Grande do Sul, Porto Alegre, Brazil; 4 Graduate Program in Medical Sciences – Endocrinology, Federal University of Rio Grande do Sul, Porto Alegre, Brazil; 5 Department of Life Sciences, University of Santa Cruz do Sul, Santa Cruz do Sul, Brazil; 6 Centro Universitario Regional Noreste, Universidad de La República, Rivera, Uruguay; 7 Department of Sports and Computer Science, Universidad Pablo de Olavide, Seville, Spain; 8 Graduate Program in Child & Adolescent Health, Federal University of Rio Grande do Sul, Porto Alegre, Brazil

**Keywords:** Genetic predisposition, Adiposity, Child, Young, FTO polymorphism

## Abstract

**Background:**

The behavior of anthropometrics and the relationship with genetic factors through a long-term perspective should be better explored. This study aims to verify the odds of maintaining the nutritional status classification after three years, according to the rs9939609 polymorphism (FTO gene).

**Methods:**

It was a retrospective longitudinal study with 355 schoolchildren (7–17 years). Body mass index, body-fat percentage (BF%), and waist circumference (WC) were measured at baseline and follow-up. The FTO gene was evaluated from blood collection and genotyping performed by real-time polymerase chain reaction. Odds ratios and 95% confidence intervals were calculated.

**Results:**

For those homozygous with the A allele, the odds of being at less favorable classification at follow-up were 2.29 (1.24; 4.22) and 4.05 (2.08; 7.86) times higher than expected for BF% and WC, respectively, whereas the odds of being in the more favorable classification at follow-up were 0.34 (0.12; 0.93) and 0.11 (0.01; 0.78) for BF% and WC, respectively. The odds of being at less favorable classification were higher for AA carriers with less favorable classification at baseline for BF% and WC compared to AT and TT carriers.

**Conclusions:**

Schoolchildren with a genetic predisposition to obesity and unfavorable anthropometric profile at baseline had more chances of maintaining their nutritional status after three years of follow-up.

**Supplementary Information:**

The online version contains supplementary material available at 10.1186/s12887-023-03846-0.

## Background

Childhood obesity is a current issue, considering its high prevalence and relation to comorbidities during adulthood [1–3]. Even though obesity is a multifactorial issue, genetic factors are responsible for approximately 40–70% of adult obesity [4]. The rs9939609 polymorphism, on the fat mass and obesity-associated gene (FTO), has been continuously related to obesity, being the A allele the risk factor. The association between FTO gene polymorphisms and obesity was first established in European populations, later being tested and confirmed with Asians, but to a lesser extent [5]. In Africans, the relationship remains uncertain [5]. For children and adolescents, this is evident in a meta-analysis that identified a higher risk of overweight and obesity for risk genotype in polymorphisms of the FTO gene, in Caucasians (OR: 1.38; 95% CI: 1.29 to 1.49) and Amerindians (OR: 1.22; 95% CI: 1.04 to 1.43), but not in the only study on Africans (OR: 1.05; 95% CI: 0.91 to 1.21) [6]. In another meta-analysis [7], which focused on rs9939609 polymorphism, similar results were detected, with findings by ethnicity limited because it included two studies, clustering Amerindians and Asians.

Studies in South America are still a minority when compared to other continents, which makes it difficult to generalize the findings for this population, even though considering the different ethnicities and miscegenation in some countries [8]. In Brazil, a longitudinal study of the North Region, with a predominance of brown and black children, showed an annual increase in body mass index (BMI) between 0.07 to 0.08 kg/m^2^/year and 0.03 *Z*/year in BMI-for-age *Z* score, for each A allele of polymorphism rs9939609 (FTO gene) [9]. On the other hand, Pereira et al. [10] did not identify an association between excess weight and the same polymorphism in children from the Southeast. Previous findings using the same children and adolescent population within the present study also exhibited an association between the FTO gene and obesity [11]. It is important to highlight that in the Brazilian historical context, there is an ethnic heterogeneity, due to miscegenation, mainly among Amerindians, Europeans, and sub-Saharan Africans [12]. Moreover, color/race self-identification is used, with categories formed belonging to a cultural identity recognized by physical characteristics [13]. Considering this information, Alves-Silva et al. [12] identified, through mtDNA, that over half of subjects who call themselves white have Amerindian or African matrilineal genetic contributions (33% and 28%, respectively), with regional differences. This characteristic reflects the frequency of polymorphisms and may be implicated in the divergent findings among Brazilian children and adolescents.

Thus, it is necessary to expand the evidence concerning the anthropometric status and the relationship with genetic factors, in ethnically mixed populations, with useful markers in clinical and epidemiological practice. Therefore, the aim was to verify the odds of maintaining the nutritional status classification after three years, according to the rs9939609 polymorphism (FTO gene) of Brazilian schoolchildren.

## Methods

### Design and participants

This is a retrospective longitudinal study conducted with children and adolescents aged 7 to 17 years of both sexes, who were enrolled in schools of Santa Cruz do Sul—Rio Grande do Sul, Brazil. Schoolchildren who participated in projects "Evaluation of biochemical health indicators of schoolchildren using infrared spectroscopy, polymorphisms, oral health and lifestyle factors: a study in Santa Cruz do Sul—Phase II" during 2011 and 2012, and "Schoolchildren’s Health—Phase III" during 2014 and 2015 were selected. The evaluation in 2011/12 was considered the baseline, and 2014/15 was the follow-up. The research was previously appreciated and approved by the Committee of Ethics in Research with Human Beings of the University of Santa Cruz do Sul (protocol number 1.836.983) and followed resolution 466/2012 of the National Council of Health in Brazil. The parents or legal guardians of schoolchildren of 7 to 17 years signed written free and informed consent forms, one copy being given to the subject and the other to the researcher.

**Fig. 1:**
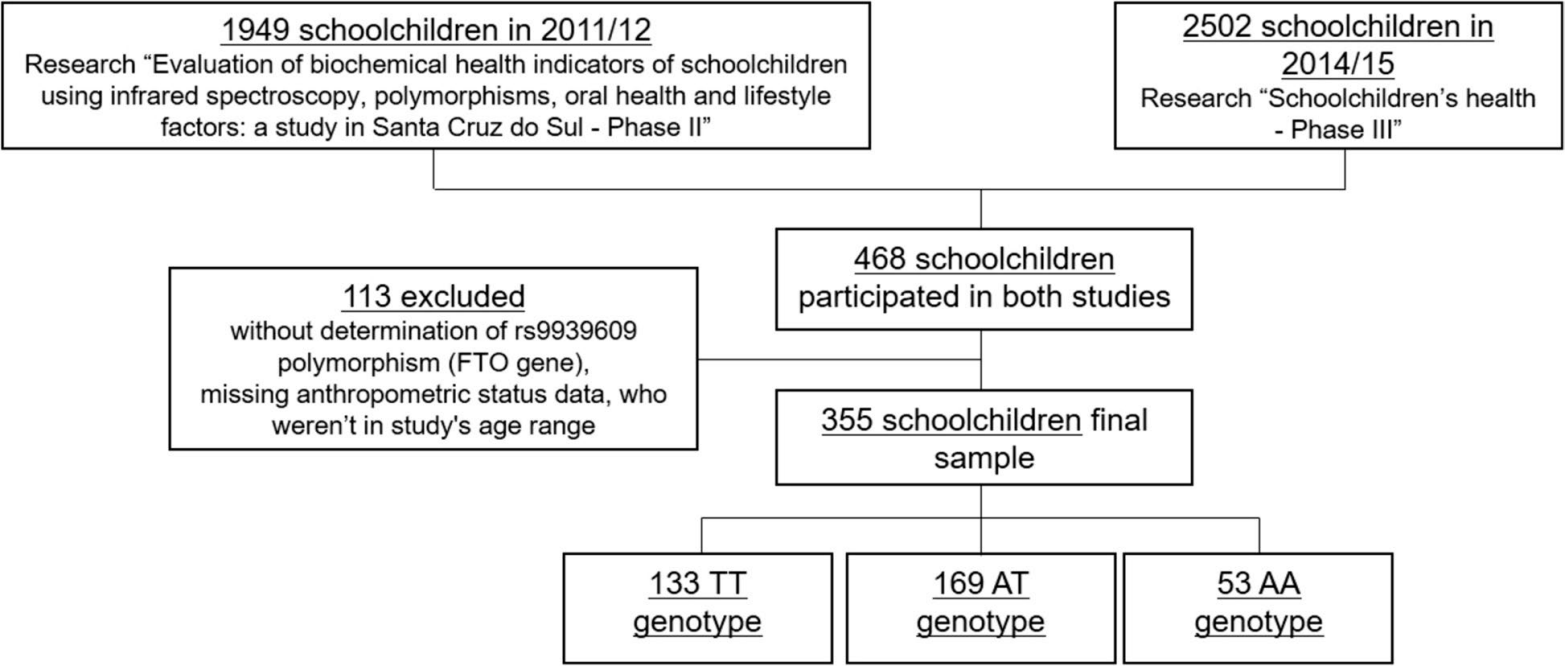
Flow diagram for final sample composition of schoolchildren in a city in southern Brazil

Both surveys considered private and public schools, elementary and high schools from the central, north, south, east, and west zones. Children and adolescents with complete demographic and socioeconomic data, anthropometric status, and rs9939609 polymorphism (FTO gene) were identified (Fig. 1). The minimum sample size needed for a 95% confidence interval of the estimated value of the proportion and a margin of error of 5% is 378. However, the sample size within the present study is 355, which represents a margin of error of 5.16%. [14].

### Data collection

The equipment used for assessments was the same at baseline and follow-up. Additionally, a trained team of professionals conducted the evaluations to ensure test standardization.

The identification data (i.e. gender, and skin color) were self-reported by students during interviews. It was complemented by the school region (urban and rural) and the school network (state and municipal). Age was confirmed by birthdate offered by the school.

Measures of total and central obesity were considered for analysis. Concerning total obesity, BMI and body fat percentage (BF%) were used. BMI was evaluated by dividing weight (kg) by squared height (m) using an anthropometric scale with a coupled stadiometer. The participants were instructed to wear light clothes and should be barefoot during the test. The percentiles of the World Health Organization [15] were used for classification: < P85 was considered eutrophy, whereas BMI ≥ P85 was considered as having excess weight. Triceps and subscapular skinfolds were assessed using Lange® compass. Slaughter et al. [16] equation was used to estimate BF%. The Lohman criteria [17] was used for classification, with a subsequent dichotomization into desirable (very low, low, and optimal) and undesirable (moderately high, high, and very high). Central obesity assessment was assessed using the waist circumference (WC) measurement at the central point between the iliac crest and last rib, classified according to Fernández et al. [18] criteria.

The rs9939609 polymorphism (FTO gene) was evaluated from blood collection of the brachial vein, considering the genotypes TT, AT, and AA. DNA extraction was performed by the salting out method [19], from EDTA whole blood samples. Genotyping was performed by real-time polymerase chain reaction (PCR) (StepOne Plus, Applied Biosystems, CA, USA) with probes TaqMan type (Applied Biosystems, CA, USA). Details of the procedures were described previously [20].

### Statistical methods

The Statistical Package for the Social Sciences (SPSS, version 23.0 IBM, Armonk, NY) software was used for all statistical analysis. The Chi-square test was applied to compare the observed and expected values ​​for each genotype. Data were in Hardy–Weinberg Equilibrium (*p* > 0.05). Shapiro–Wilk test was used to test data normality. A descriptive analysis was performed to describe the subjects at baseline and follow-up according to rs9939609 polymorphism (FTO gene). The Chi-square test and the Kruskal–Wallis test were used to compare frequencies and continuous differences, respectively, in both periods. *P*-values < 0.05 were considered statistically significant. A proportion was calculated for positive results of participants classified on a respective anthropometric classification divided by all participants stratified by rs9939609 polymorphism (FTO gene). Odds ratios and 95% confidence intervals for the number of participants on a risk anthropometric classification at baseline were calculated for how many continued at the same or changed to another anthropometric classification in the follow-up. Expected numbers were calculated based on a random distribution of change in anthropometric risk factors.

## Results

From the total sample, 62.5% presented an A allele (AT: 47.6%; AA: 14.9%). At baseline, children and adolescents had between 7 to 15 years of age (median: 10 years and interquartile range: 8–11 years), and 9 to 17 years of age (median: 12 years; interquartile range: 11–14 years) at follow-up, with no differences between groups (*p* ≥ 0.05). The genotype groups were homogeneous for demographic and anthropometric characteristics at baseline and in the follow-up, as described in Table 1.

**Table 1:** Descriptive characteristics according to rs9939609 polymorphism (FTO gene) at baseline and in the follow-up

	**2011/12 (Baseline)**			***p***	**2014/15 (Follow-up)**			***p***
	**TT** **(*n* = 133)**	**AT** **(*n* = 169)**	**AA** **(*n* = 53)**		**TT** **(*n* = 133)**	**AT** **(*n* = 169)**	**AA** **(*n* = 53)**	
Demographic		**n (%)**				**n (%)**		
Sex								
Male	58 (43.6)	74 (43.8)	24 (45.3)	0.977^a^	N/A	N/A	N/A	N/A
Female	75 (56.4)	95 (56.2)	29 (54.7)		N/A	N/A	N/A	
Skin color								
White	102 (76.7)	137 (81.1)	43 (81.1)	0.612^a^	N/A	N/A	N/A	N/A
Other	31 (23.3)	32 (18.9)	10 (18.9)		N/A	N/A	N/A	
School region								
Urban	81 (60.9)	84 (49.7)	26 (49.1)	0.115 ^a^	82 (61.7)	83 (49.1)	25 (47.2)	0.057^a^
Rural	52 (39.1)	85 (50.3)	27 (50.9)		51 (38.3)	86 (50.9)	28 (52.8)	
School network								
Municipal	61 (45.9)	73 (43.2)	27 (50.9)	0.607^a^	57 (42.9)	67 (39.6)	25 (47.2)	0.605^a^
State	72 (54.1)	96 (56.8)	26 (49.1)		76 (57.1)	102 (60.4)	28 (52.8)	
Anthropometric		**Median** **(Q1; Q3)**		***p***		**Median** **(Q1; Q3)**		***p***
Body mass index (kg/m^2^)	18.0 (15.9; 21.4)	18.5 (16.7; 21.4)	17.7 (15.8; 20.2)	0.112^b^	20.4 (18.0; 23.1)	20.5 (18.5; 23.8)	20.3 (17.1; 22.3)	0.357^b^
Body fat percentage (%)	21.6 (16.4; 27.5)	21.9 (17.5; 27.5)	20.0 (15.6; 25.7)	0.274^b^	20.2 (16.4; 26.0)	20.5 (14.6; 27.0)	21.2 (14.2; 24.7)	0.782^b^
Waist circumference (cm)	60.3 (55.5; 70.0)	63.0 (58.0; 69.8)	62.0 (55.0; 68.0)	0.099^b^	68.0 (62.0; 74.2)	69.0 (63.3; 77.0)	68.9 (61.2; 73.7)	0.297^b^

Data are expressed as absolute values for categorical variables or as the median and interquartile range (Q1 to Q3) for continuous variables; The *p*-value was calculated using the Chi-square test for for categorical variables or the Kruskal-Wallis test for continuous variables; significant differences for *p*<0.05;

*FTO* Fat mass and obesity-associated, *kg* Kilograms, *m* Meters, *cm* centimeters

^a^chi-square test

^b^Kruskal–Wallis test

**Fig. 2:**
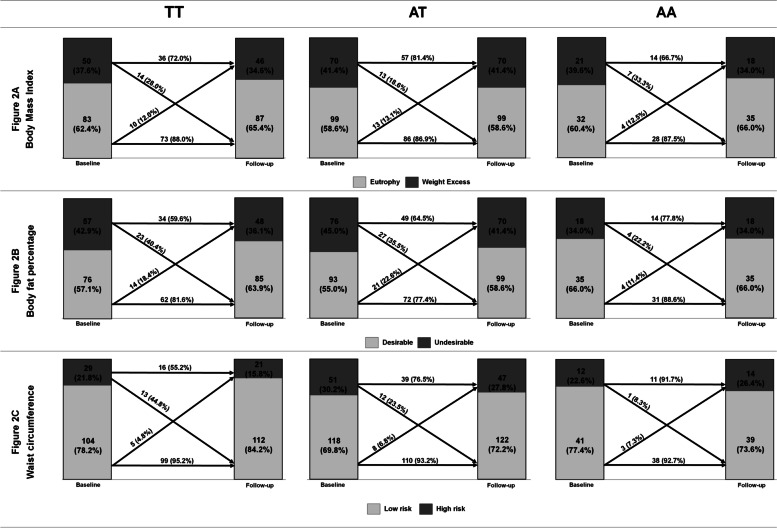
Descriptive maintenance of the anthropometric classification from baseline to follow-up according to rs9939609 polymorphism

Descriptive changes across the three years were described in Fig. 2. Participants with the AA genotype exhibited higher descriptive percentages of maintenance in the less favorable nutritional status from baseline to follow-up for undesirable BF% (77.8%) and high-risk waist circumference (91.7%).

**Fig. 3:**
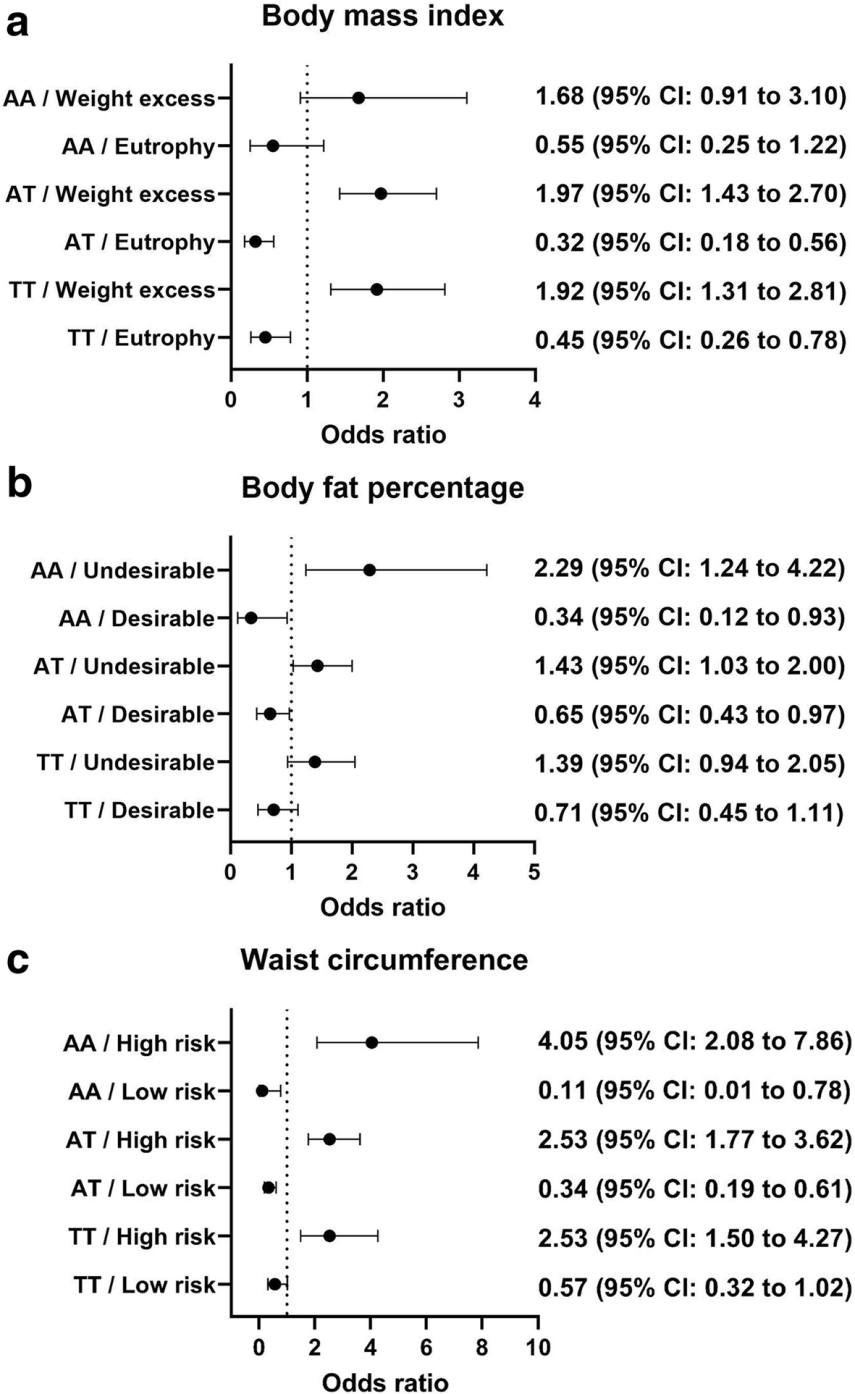
**a** BMI classification from baseline to follow-up for participants with excess weight in the baseline. **b** Body fat percentage classification from baseline to follow-up for participants with undesirable levels in the baseline. **c** Waist circumference classification from baseline to follow-up for participants with high risk in the baseline

Figure 3 (a, b, c) presents odds ratios and 95% confidence intervals for the number of participants on a risk nutritional status at baseline who maintained the classification or changed to another classification in the follow-up for BMI, BF%, and WC, respectively (see Additional file 1 for all data). For those who had the AA genotype and were at the less favorable nutritional status, the odds of staying at this classification were 2.29 (95% CI: 1.24 to 4.22) and 4.05 (95% CI: 2.08 to 7.86) times more than expected for BF% and WC, respectively, whereas the odds of being classified in the more favorable classification in the follow-up were 0.34 (95% CI: 0.12 to 0.93) and 0.11 (95% CI: 0.01 to 0.78) for BF% and WC, respectively. No significant odds ratios were observed for staying at the less favorable (OR: 1.68; 95% CI: 0.91 to 3.10) or moving to the more favorable (OR: 0.55; 95% CI: 0.25 to 1.22) BMI classification.

## Discussion

The main findings indicated that for children and adolescents with the AA genotype, the odds of staying at a less favorable anthropometric classification in the follow-up were higher than expected for BF% and WC classifications. Thus, it is suggested that genetic predisposition to obesity increases the chances of children and adolescents maintaining an unfavorable anthropometric profile after three years of follow-up.

The literature has previously assessed the association between the FTO gene and adiposity. A Finnish study evaluated the rs9939609 polymorphism (FTO gene) from the first year of life. The authors found that between 7 and 15 years of age an association of homozygote A allele was observed in BMI values when compared to those with at least one T allele [21]. In China, a meta-analysis study observed that rs9930609 polymorphism (FTO gene) presented a relationship with obesity in children and adolescents [22]. In addition, another meta-analysis study found that children and adolescents with AA or AT genotype had a high risk of developing overweight and obesity. However, an inverse association was observed when there was the presence of the T allele (TT or AT genotype) [8]. The finding of the present study partially agrees with the aforementioned studies since there was an increase in the risk of weight excess assessed by BMI for the AT genotype, but not in the AA genotype.

In Brazilian infant and juvenile population studies, results are divergent. It is important to note that obesity in Brazil is relevant since there is an estimate of approximately 40% of Brazilian children and adolescents with excess weight/obesity [23]. Also, Brazil is among the ten countries concentrating more than 50% of obesity around the world [24]. Secular tendency indicates an increase in the occurrence of overweight between 1975 to 2016 [25]. Our findings showed that individuals with the less favorable classification of %BF and WC, in addition to presenting the AA genotype, were more likely to remain in the same classification. The absence of association was reported by Pereira et al. [10], who evaluated AKT1, FTO, and AKTIP polymorphisms. When following up on 348 children, Silva et al. [2] identified two milestones: higher BMI Z -scores for the AA genotype at age 4 and increased subcutaneous fat at age 8. Another study performed in Brazil observed that children and adolescents with the presence of the risk allele of rs9939609 polymorphism had high abdominal adiposity [11]. The scientific evidence on the contribution of polymorphism investigated here is mainly related to Caucasian populations [7], and the Brazilian ethnic composition allows regional differences for association with the outcome of obesity [10]. In this sense, our sample consisted of 79.4% of participants who described themselves as white and, it is important to reinforce that a significant percentage of matrilineal genetic contribution from other ethnicities was identified in self-declared white. This phenomenon is due to relevant historical aspects in the construction of the identity of Brazilians [12].

Moreover, it is important to consider that obesity is a complex and multifactorial condition. Therefore, in addition to genetic susceptibility, environmental interaction has been widely investigated. Thus, the results of the present study can be explained from two perspectives: first, the genetic predisposition to obesity seems to increase the risk of being overweight, once the FTO polymorphism seems to act directly in adipogenesis, as well as in the hypothalamic function of energy homeostasis [26, 27], factors that are associated with increased body weight; second, behavioral, environmental and social factors can influence in the development of obesity, like the presence of unhealthy lifestyle and urbanization process allied with technological advancement that is associated with low physical activity levels and high screen time of children and adolescents [28, 29].

Therefore, it is important to monitor the pediatric population with a genetic predisposition to obesity. For this, it is necessary to encourage this population to adopt healthy habits to prevent overweight and obesity [8, 30], once the odds of children and adolescents with the risk allele remaining with adiposity over time is high. Moreover, the adoption of a healthy lifestyle, like a balanced diet, and the practice of physical exercise seems to be able to cause modular effects of the FTO polymorphism [8, 31].

Some limitations must be acknowledged. We did not consider possible determinants of body composition, such as food consumption, time spent practicing physical exercise, time in sedentary behavior, and other lifestyle habits that can influence the association between genetic predisposition and anthropometric measurements. In addition, there are other simple nucleotide polymorphisms in the FTO gene, which may be associated with the risk of obesity [21]. Also, as this is a retrospective study, some information, such as pubertal development, wasn't collected at baseline. However, the present study deepens the theme in an ethnically mixed population, in an age group that included children and adolescents, with the use of measures of total and central obesity. Still, it contemplated three years, amplifying the information on risk allele influence at different ages. In this sense, our findings highlight the importance of monitoring individuals with the risk allele of the rs9939609 polymorphism (FTO gene) since childhood, especially those with excess body adiposity, to reduce the deleterious effects caused by obesity on health since higher adiposity levels seem to track from early childhood to adolescence (especially for AA genotype carriers as demonstrated in the present study) and further to adulthood [32].

## Conclusions

Schoolchildren with a genetic predisposition to obesity had more chances of maintaining their low nutritional status after three years of follow-up, especially in those with an unfavorable anthropometric profile at baseline.

## Supplementary Information

**Additional file 1:** **Supplementary Table 1.** Odds ratio of the anthropometric classification from baseline to follow-up for all participants.

## Data Availability

The database used and analyzed in the present study is not publicly available as its information may compromise the participants' privacy and consent involved in the research. However, the data are available from the corresponding author, upon request.
