# Supplementary material for: The genetic predisposition increases the chances of schoolchildren maintaining higher adiposity levels after three years
Source: BMC Pediatr. 2023 Feb 3;23:57. doi: 10.1186/s12887-023-03846-0 (PMC9896808; doi:10.1186/s12887-023-03846-0)
Supplement: Supplementary file 1 — Additional file 1: Supplementary Table 1. Odds ratio of the anthropometric classification from baseline to follow-up for all participants. [file 12887_2023_3846_MOESM1_ESM.docx]

**Supplementary Table 1.** Odds ratio of the anthropometric classification from baseline to follow-up for all participants.

|  | **TT** | | | **AT** | | | **AA** | | |
| --- | --- | --- | --- | --- | --- | --- | --- | --- | --- |
|  | **Baseline** | **Follow-up** | | **Baseline** | **Follow-up** | | **Baseline** | **Follow-up** | |
|  | **P**  **(95% CI)** | **OR**  **(95% CI)** | **OR**  **(95% CI)** | **P**  **(95% CI)** | **OR**  **(95% CI)** | **OR**  **(95% CI)** | **P**  **(95% CI)** | **OR**  **(95% CI)** | **OR**  **(95% CI)** |
| Body Mass Index | | Eutrophy | Weight excess |  | Eutrophy | Weight excess |  | Eutrophy | Weight excess |
| Eutrophy | 0.62  (0.54; 0.71) | 1.41  (1.01; 1.98) | 0.32  (0.17; 0.61) | 0.59  (0.51; 0.66) | 1.48  (1.10; 2.00) | 0.32  (0.18; 0.56) | 0.60  (0.46; 0.74) | 1.45  (0.85; 2.49) | 0.32  (0.11; 0.87) |
| Weight   excess | 0.38  (0.29; 0.46) | 0.45  (0.26; 0.78) | 1.92  (1.31; 2.81) | 0.41  (0.34; 0.49) | 0.32  (0.18; 0.56) | 1.97  (1.43; 2.70) | 0.40  (0.27; 0.54) | 0.55  (0.25; 1.22) | 1.68  (0.91; 3.10) |
| Body fat percentage | | Desirable | Undesirable |  | Desirable | Undesirable |  | Desirable | Undesirable |
| Desirable | 0.57  (0.48; 0.66) | 1.43  (1.02; 2.01) | 0.43  (0.25; 0.75) | 0.55  (0.47; 0.63) | 1.41  (1.04; 1.91) | 0.50  (0.32; 0.79) | 0.66  (0.52; 0.79) | 1.34  (0.78; 2.32) | 0.34  (0.12; 0.93) |
| Undesirable | 0.43  (0.34; 0.52) | 0.71  (0.45; 1.11) | 1.39  (0.94; 2.05) | 0.45  (0.37; 0.53) | 0.65  (0.43; 0.97) | 1.43  (1.03; 2.00) | 0.34  (0.22; 0.48) | 0.34  (0.12; 0.93) | 2.29  (1.24; 4.22) |
| Waist circumference | | Low risk | High risk |  | Low risk | High risk |  | Low risk | High risk |
| Low risk | 0.78  (0.70; 0.85) | 1.22  (0.82; 1.80) | 0.22  (0.09; 0.54) | 0.70  (0.62; 0.77) | 1.34  (0.97; 1.83) | 0.22  (0.11; 0.46) | 0.77  (0.64; 0.88) | 1.20  (0.66; 2.18) | 0.32  (0.10; 1.04) |
| High risk | 0.22  (0.15; 0.30) | 0.57  (0.32; 1.02) | 2.53  (1.50; 4.27) | 0.30  (0.23; 0.38) | 0.34  (0.19; 0.61) | 2.53  (1.77; 3.62) | 0.23  (0.12; 0.36) | 0.11  (0.01; 0.78) | 4.05  (2.08; 7.86) |

P: The proportion of positive results of participants classified on a respective anthropometric classification divided by all participants stratified by rs9939609 polymorphism (FTO gene) (TT=133; AA=53; or AT=169); 95% CI: 95% confidence interval; OR: Odds ratio of how many participants were at a respective anthropometric classification in the follow-up divided by the expected number of being at this classification in the follow-up if anthropometric risk factors changed randomly.
